# Dehydroepiandrosterone and Its CYP7B1 Metabolite 7α-Hydroxydehydroepiandrosterone Regulates 11β-Hydroxysteroid Dehydrogenase 1 Directions in Rat Leydig Cells

**DOI:** 10.3389/fendo.2019.00886

**Published:** 2020-01-24

**Authors:** Qiqi Zhu, Yaoyao Dong, Xiaoheng Li, Chaobo Ni, Tongliang Huang, Jianliang Sun, Ren-Shan Ge

**Affiliations:** ^1^Department of Anesthesiology, The Second Affiliated Hospital and Yuying Children's Hospital of Wenzhou Medical University, Wenzhou, China; ^2^Department of Pharmacy, The Second Affiliated Hospital and Yuying Children's Hospital of Wenzhou Medical University, Wenzhou, China; ^3^Department of Anesthesia, Hangzhou Hospital Affiliated to Zhejiang University, Hangzhou First People's Hospital, Hangzhou, China

**Keywords:** steroids, dehydroepiandrosterone, 7α-hydroxydehydroepiandrosterone, leydig cells, CYP7B1

## Abstract

**Background:** The purpose of this study was to investigate cytochrome P450-7B1 (CYP7B1) in the human and rat testes to regulate 11β-hydroxysteroid dehydrogenase 1 (11β-HSD1) activity. We hypothesized that dehydroepiandrosterone (DHEA) and its product 7α-hydroxydehydroepiandrosterone (7αOHD) after catalysis of CYP7B1 played a critical role in driving the direction of 11β-HSD1, because 7αOHD is an alternative substrate for 11β-HSD1.

**Methods:** We examined the influence of DHEA and 7αOHD on 11β-HSD1 activities in both intact Leydig cells and microsomes using radioactive substrates and identified the location of CYP7B1 in Leydig cells using immunohistochemical staining, Western blot, and qPCR.

**Results:** We found that DHEA stimulated 11β-HSD1 oxidase activity in intact cells (EC_50_ = 0.97 ± 0.11 μM) and inhibited its reductase activity (IC_50_ = 1.04 ± 0.06 μM). In microsomes, DHEA was a competitive inhibitor of the reductase activity. The 11β-HSD1 oxidase activity in intact cells was inhibited by 7αOHD (IC_50_ = 1.18 ± 0.12 μM), and the reductase activity was enhanced (EC_50_ = 0.7 ± 0.04 μM). 7αOHD was a competitive inhibitor of 11β-HSD1 oxidase. CYP7B1 was present in rat Leydig cells, as shown by immunohistochemistry, Western blotting, and qPCR analysis.

**Conclusion:** Our results are consistent with a conclusion that DHEA in the circulation driving 11β-HSD1 toward an oxidase in Leydig cells mainly through inhibiting the reductase of the enzyme, while 7αOHD (CYP7B1 catalytic product of DHEA) drives the enzyme toward the opposite direction.

## Introduction

11β-Hydroxysteroid dehydrogenases are a set of enzymes that catalyzes interconversion of active glucocorticoid (GC), cortisol (humans) or corticosterone (CORT, rodents), and inert GC, cortisone (humans) and 11-dehydrocorticosterone (DHC, rodents). There are two isoforms: type I (11β-HSD1) and type II (11β-HSD2) (1–4). 11β-HSD1 is an NADP^+^/NADPH-dependent bidirectional enzyme and is primarily present in liver and testis (5, 6). 11β-HSD2 is an unidirectional NAD^+^-dependent oxidase and is mainly present in kidney and colon (7).

The direction of 11β-HSD1 is influenced by NADP^+^/NADPH ratio in the smooth endoplasmic reticulum (SER). 11β-HSD1 is a membrane binding enzyme with cofactor-binding domain facing SER lumen (8–10). In liver cells, 11β-HSD1 co-localizes and shares NADP^+^/NADPH with hexose-6-phosphate dehydrogenase (H6PDH), which stays in SER lumen (11). H6PDH uses substrate, glucose 6-phosphate, which is transported into SER lumen by glucose 6-phosphate transporter (11). The membrane of SER is impermeable to NADP(H). The abundant supply of glucose 6-phosphate to H6PDH generates sufficient NADPH from NADP^+^, increasing NADPH/NADP^+^ ratio and driving 11β-HSD1 toward as a reductase in liver cells, which generates NADP^+^ and 11β-hydroxysteroids.

As a major component of the pentose phosphate pathway, the cytosolic glucose-6-phosphase dehydrogenase, which accounts for 95% of NADPH amount generated in the whole cell, does not seem to affect the direction of 11β-HSD1 catalysis (12). This is supported by the fact that depletion of glucose-6-phosphase dehydrogenase by RNA silencing does not change the direction of 11β-HSD1 catalysis (12). In contrast, H6PDH, which accounts for only 5% of NADPH production, causes 11β-HSD1 to act as a reductase in liver cells, because these two enzymes and the co-factors are compartmentalized in SER lumen. Therefore, when H6PDH was knocked out, 11β-HSD1 in the liver became a primary oxidase (13).

However, 11β-HSD1 direction in other cell types may not be regulated by H6PDH (14). Unlike liver cells, 11β-HSD1 in rat adult Leydig cells (ALCs) are primarily an oxidase with comparable reductase activity (6). Although there is small amount of 11β-HSD2, which is only about 1/1000 of 11β-HSD1 in rat ALCs (15), its low abundance does not explain directionality of 11β-HSD in this cell type. Human Leydig cells also express 11β-HSD1 and 11β-HSD2 and Leydig cells can catalyze the interconversion of 11β-hydroxysteroids and 11keto-steroids (16). Other factors may also drive 11β-HSD1 direction in rat and human ALCs. These factors may include steroids from adrenals or steroids generated locally. In this regard, 11β-HSD1 oxidase activity was significantly reduced in ALCs isolated from adrenalectomized rats when compared to that in cells in adrenal-intact controls (17). Therefore, adrenal-secreted steroids might be involved in maintaining the direction of 11β-HSD1 in rat ALCs. Dehydroepiandrosterone (DHEA) is a steroid mainly synthesized by human adrenal glands and testes as a precursor for cortisol and testosterone, and is one of the most abundant steroids in human blood. DHEA can be considered a candidate regulator of 11β-HSD1 direction because of high circulating levels and structural resemblance to the substrate of 11β-HSD1.

DHEA is also the substrate for SER cytochrome P450-dependent enzyme CYP7B1, and which is expressed in various tissues, including brain, prostate, and testis (18, 19). CYP7B1 catalyzes the conversion of DHEA into 7α-hydroxydehydroepiandrosterone (7αOHD) and generates NADP^+^. Therefore, the metabolic coupling with CYP17B1 is also possible whereby NADP^+^ drives 11β-HSD1 oxidase activity. 7αOHD may be a regulator because it is a substrate of 11β-HSD1. The epimerase activity of 11β-HSD1 converts 7αOHD into 7β-dehydroepiandrosterone (7βOHD) (20–22). However, human, pig, and rat 11β-HSD1 also catalyze 7αOHD using NADP^+^ as cofactor into 7keto-DHEA (7KD) and NADPH (23). We hypothesize that DHEA in the circulation may affect 11β-HSD1 direction in three ways: by direct stimulation of the enzyme, or through metabolic coupling with CYP7B1, or by affecting the enzyme via the metabolite, 7αOHD. The goal of the present study was to investigate each of these possibilities.

## Materials and Methods

### Chemicals and Animals

CORT, DHEA, and 7αOHD were purchased from Steraloids (Wilton, NH). [1,2-^3^H] Corticosterone (^3^H-CORT), specific activity 40 Ci/mmol, was purchased from Dupont-New England Nuclear (Boston, MA). [1,2-^3^H]11-dehydrocorticosterone (^3^H-DHC) was prepared from labeled ^3^H-CORT as described earlier (24).

### Animals

Male Sprague-Dawley rats (from 2 to 90 days old) were purchased from Shanghai Laboratory Animal Co. Ltd. (Shanghai, China). Rats were sacrificed at postnatal days 2, 7, 14, 21, 28, 35, and 90 by asphyxiation with CO_2_. One testis per rat was removed and frozen in liquid nitrogen for RNA extraction. The other testis was punched 5 holes using a needle and was fixed by Bouin's solution. The animal protocol was approved by the Institutional Animal Care and Use Committee of the Wenzhou Medical University.

### Cell Isolation

Testes were processed for purification of Leydig cells. The progenitor (PLC), immature (ILC), and adult Leydig cells (ALC) were purified from rat testes at 21, 35, and 90 days postpartum, as described previously (25, 26). In brief, forty 21-day-old (for PLCs), eighteen 35-day-old (for ILCs), and six 90-day-old (for ALCs) rats were sacrificed, followed by removal and decapsulation of testes. Then, the testes were digested with collagenase and cells were separated by Percoll density gradient centrifugation. Purities of Leydig cell fractions were evaluated by histochemical staining of 3β-hydroxysteroid dehydrogenase (3β-HSD), with 0.4 mM etiocholanolone as an enzyme substrate (27). At least six isolations per cell types were performed.

### Preparation of Microsomal Protein

The microsomes from ALCs were prepared as described previously (6). In brief, ALCs were homogenized in cold 0.01M PBS, pH 7.2, containing 0.25 M sucrose. Subsequently, the homogenates were centrifuged at 700 × g for 30 min and the supernatants were transferred to other tubes followed by centrifugation at 10,000 × g for 30 min. The resultant supernatants were centrifuged twice at 105,000 × g for 1 h. The pellets were resuspended and the protein contents were determined by Bio-Rad Protein Assay Kit (cat# 500–0006, Bio-Rad, Hercules, CA). Microsomes were used to assay 11β-HSD1 oxidoreductase activities.

### Primer Selection

All primers in this study were chosen using a sequence analysis software package (Primer 3, Whitehead Institute for Biomedical Research, Cambridge, MA) following guidelines for internal stability. Forward and reverse primers were in different exons to minimize the effects of possible DNA contamination. The primers for *Cyp7b1* were: 5'-GAAGTCCTGCGTGACGAAAT-3' (forward); 5'-CCTCAGAACCTCAAGAATAGCG-3' (reverse); and the size of PCR product is 138 bp. For the internal standard, primers to ribosomal protein S16 (*Rps16*) were used, as described previously (28).

### Real-Time PCR

PCR was carried out in a 25 μl volume using a 96-well plate format using the SYBR Green PCR Core Reagents purchased from Applied Biosystems (Foster City, CA). Primer titration was performed and the concentration of 300 nM was selected. Fluorescence was detected in real-time on an ABI 5700 system (PE Applied Biosystems). The mRNA levels were measured by a standard curve method and the levels were normalized to *Rps16*, the internal control. Each sample was run in duplicate, in parallel with no template controls.

### Immunofluoroscent Staining

Immunofluoroscent staining of CYP7B1 was performed on isolated ALCs using cells grown on 12-well microscope cover glasses. Cells were fixed with 4% formaldehyde, washed with PBS, and permeabilized with 0.1% Triton X100 in PBS plus 10% normal serum. Nonspecific binding was blocked by incubation with 10% normal serum before addition of the primary antibody. Cells were incubated with goat polyclonal CYP7B1 antibody (Santa Cruz Biotechnology, Santa Cruz, CA) for 1 h at room temperature. Cells were then incubated with Alexa488-conjugated second antibody for 1 h. The cells were counterstained with DAPI and mounted onto glass microscope slides, and cover-slipped. The slides were examined under a Nikon fluorescence microscope with a filter suitable for detecting the fluorescence of green color.

### Immunohistochemical Staining

Testes were dehydrated in ethanol and xylene and embedded in paraffin for immunological analysis. 6 μm-thick transverse sections were prepared and mounted on glass slides. Avidin–biotin immunostaining was performed using a Vector kit (Burlingame, CA) according to the manufacturer's instructions. Antigen retrieval was carried out by boiling for 10 min in 10 mM (pH 6.0) citrate buffer, and endogenous peroxidase was blocked with 0.5% H_2_O_2_ in methanol for 30 min. The sections were then incubated with goat polyclonal CYP7B1 antibody (1:200, Santa Cruz Biotechnology, Santa Cruz, CA) for 1 h at room temperature. The antibody-antigen complexes were visualized with diaminobenzidine alone, resulting in brown cytoplasmic staining in positively labeled cells. The sections were counterstained with Mayer hematoxylin, dehydrated in graded concentrations of alcohol, and cover-slipped with resin (Permount, SP15-100; Fisher Scientific).

### Western Blotting

Western blotting was performed as previously described (29). Briefly, rat PLC, ILC, and ALC homogenates (30 μg protein each sample) were electrophoresed on 10% polyacrylamide gel containing sodium dodecyl sulfate. After electrophoresis, proteins were transferred onto a nitrocellulose membrane, and the membrane was incubated with 5% non-fat milk for 1 h to block the non-specific binding. Then, the membrane was incubated with primary antibodies against the following antigens: CYP7B1 (Santa Cruz Biotechnology) and β-actin (ACTB, Cell Signaling Technology, Danvers, MA), which serves the internal control. The membrane was washed and incubated with a 1:5,000 dilution of second antibody conjugated to horseradish peroxidase. Immunoreactive bands were visualized by an ECL kit (Amersham, Arlington Heights, IL). The intensity of the protein was quantified using Image J software. The CYP7B1 was adjusted to ACTB.

### 11β-HSD1 Oxidase and Reductase Assay in Intact ALCs

11β-HSD1 oxidase and reductase in intact ALCs were performed as previously described (15).11β-HSD1 oxidase activity assay tubes contained 25 nM CORT spiked with 60,000 dpm [^3^H]-CORT (a concentration within the range of physiological levels of CORT). 11β-HSD1 reductase activity assay tubes contained 25 nM DHC spiked with 60,000 dpm [^3^H]-DHC. 2.5 × 10^4^ intact ALCs were added to start the reaction and the reaction was maintained in 34°C and 75 rpm shaking water bath for 30 min. The reactions were stopped by adding 2 ml ice-cold ether. The steroids were extracted, and the organic layer was dried under nitrogen. The steroids were separated chromatographically on thin layer plates in chloroform and methanol (90:10, v/v), and the radioactivity was measured using a scanning radiometer (System AR2000, Bioscan Inc., Washington, DC). The percentage conversion of CORT to DHC and DHC to CORT was calculated by dividing the radioactive counts identified as DHC (or CORT, respectively) by the total counts associated with CORT plus DHC.

### 11β-HSD1 Oxidase and Reductase Assay in Microsomes

11β-HSD1 oxidase and reductase in microsomes were performed as previously described (15). For measurements in microsomal preparations, microsomes were incubated with 25 nM [^3^H]-CORT and 0.2 mM NADP^+^ for oxidase or 25 nM [^3^H]-DHC and 0.2 mM NADPH for reductase activity in presence of different concentrations of DHEA or 7αOHD. For testing mode of action, microsomes were incubated with 2 nM to 10 μM [^3^H]-CORT and 0.2 mM NADP^+^ for oxidase or 2 nM to 10 μM [^3^H]-DHC and 0.2 mM NADPH for reductase activity. 1.5 μg of ALC microsomes were added to initiate the reaction. The reactions were stopped by adding 2 ml ice-cold ether. The steroids were extracted, and the organic layer was dried under nitrogen. The steroids were separated chromatographically on thin layer plates in chloroform and methanol (90:10, v/v), and the radioactivity was measured using a scanning radiometer (System AR2000, Bioscan Inc., Washington, DC). The percentage conversion of CORT to DHC and DHC to CORT was calculated by dividing the radioactive counts identified as DHC (or CORT, respectively) by the total counts associated with CORT plus DHC.

### Statistics

Experiments were repeated three to four times. Data were subjected to analysis by one-way ANOVA followed by *ad hoc* DUNCAN multiple comparisons testing to identify significant differences between groups when three and more groups were calculated, or by the student *t*-test when two groups were calculated. All data are expressed as means SEM. Differences were regarded as significant at ^*^P < 0.05 or ^**^P < 0.01 or ^***^P < 0.001.

## Results

### DHEA Decreases 11β-HSD1 Reductase Activity in Intact ALCs

**Figure 1:**
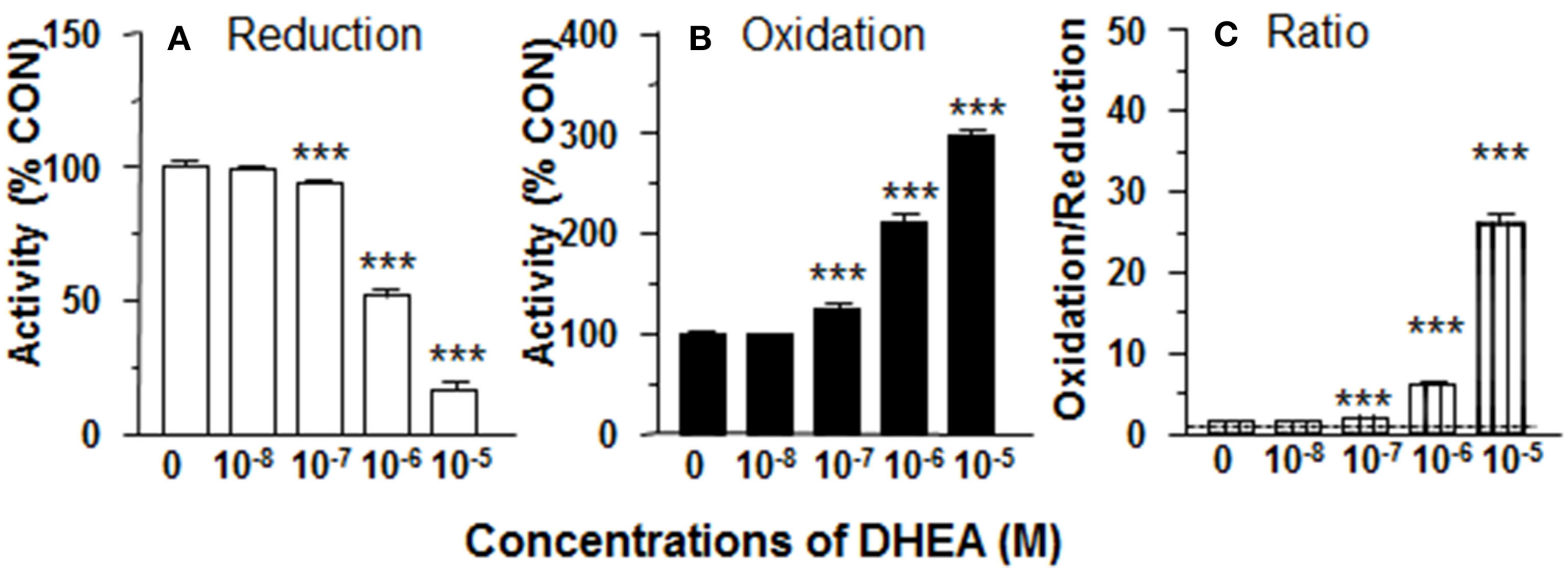
Effects of dehydroepiandrosterone (DHEA) on 11β-HSD1 oxidase and reductase activities in intact adult Leydig cells (ALCs). 2.5 × 10^4^ ALCs were cultured with 25 nM DHC (reductase, **A**) or CORT (oxidase, **B**) for 0.5 h. **(C)** is 11β-HSD1 oxidase/reductase ratio. Mean ± SEM (*n* = 4). *, **, and *** designate significant differences when compared to control (first bar in each panel) at *P* < 0.05, 0.01, and 0.001, respectively.

11β-HSD1 reductase converts DHC into CORT. We measured 11β-HSD1 reductase activity in intact ALCs in presence of DHEA (10 nM−10 μM). DHEA inhibited 11β-HSD1 reductase activity with the lowest observable effective level (LOEL) of 100 nM and it lowered 11β-HSD1 reductase activity to 12% of control at 10 μM (Figure 1A). The IC_50_ value of DHEA of inhibiting 11β-HSD1 reductase activity was 1.04 μM (Table 1). This indicates that DHEA is a potent inhibitor of 11β-HSD1 reductase in intact ALCs.

**Table 1:** EC_50_ or IC_50_ for the regulation of 11β-HSD1 oxidase and reductase activities in intact Leydig cells and microsome.

		**11β-HSD1**		**11β-HSD1**	
**Chemicals**	**EC_**50**_/IC_**50**_** **(×10^**−6**^ M)**	**Intact Leydig cells** **Oxidase**		**Microsome** **Oxidase**	
			**Reductase**		**Reductase**
DHEA	EC_50_/IC_50_	0.97 ± 0.10	1.04 ± 0.06	ND	1.29 ± 0.89
7αOHD	EC_50_/IC_50_	1.18 ± 0.12	0.7 ± 0.04	0.93 ±0.14	0.2 ± 0.01

*ND, not detected; Mean ± SEM (n = 4)*.

### DHEA Increases 11β-HSD1 Oxidase Activity in Intact ALCs

11β-HSD1 oxidase converts CORT into DHC. We measured 11β-HSD1 oxidase activity in intact ALCs in presence of DHEA (10 nM-10 μM). DHEA stimulated 11β-HSD1 oxidase activity with LOEL of 100 nM (Figure 1B). DHEA increased 11β-HSD1 oxidase activity to 300% of control at 10 μM. The EC_50_ value of DHEA of stimulating 11β-HSD1 oxidase activity was 0.97 μM (Table 1). We calculated 11β-HSD1 oxidase/reductase ratio (Figure 1C) and found that 11β-HSD1 oxidase/reductase ratio in the control was 1.6-fold conforming to the previous report (17). DHEA increased this ratio by 26-folds at 10 μM, indicating that DHEA makes 11β-HSD1 more oxidative.

### DHEA Inhibits 11β-HSD1 Reductase Activity in ALC Microsomes

**Figure 2:**
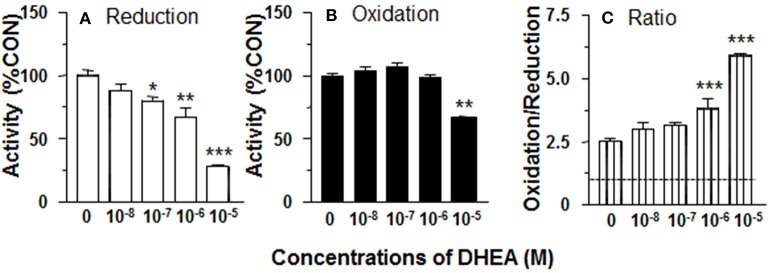
Effects of dehydroepiandrosterone (DHEA) on 11β-HSD1 oxidase and reductase activities in adult Leydig cell (ALC) microsomes. 1.5 μg ALC microsome were cultured with 25 nM DHC plus 0.2 mM NADPH (reductase, **A**) or 25 nM CORT plus 0.2 mM NADP+ (oxidase, **B**) for 0.5 h. **(C)** is 11β-HSD1 oxidase/reductase ratio. Mean ± SEM (*n* = 4). *, **, and *** designate significant differences when compared to control (first bar in each panel) at *P* < 0.05, 0.01, and 0.001, respectively.

In order to separate the potential contribution of the pentose phosphate pathway that is located in the cytosol, 11β-HSD1 reductase activity was measured in ALC microsomes in presence of DHEA (10 nM-10 μM). DHEA decreased ALC microsomal 11β-HSD1 reductase activity with LOEL of 100 nM and it lowered 11β-HSD1 reductase activity to 25% of control at 10 μM (Figure 2A). The IC_50_ value of DHEA of inhibiting 11β-HSD1 reductase activity in ALC microsomes was 1.29 μM (Table 1). This is similar to what was found in intact ALCs.

### DHEA Does Not Affect 11β-HSD1 Oxidase Activity in ALC Microsomes

11β-HSD1 oxidase activity was measured in ALC microsomes in presence of DHEA (10 nM-10 μM). DEHA did not affect microsomal 11β-HSD1 oxidase activity. Actually, DHEA also inhibited 11β-HSD1 oxidase activity at 10 μM (Figure 2B). Although DHEA did not affect 11β-HSD1 oxidase, 11β-HSD1 oxidase/reductase ratios in ALC microsomes were still significantly higher than control at 1 and 10 μM DHEA (Figure 2C). These data indicate that the stimulation of 11β-HSD1 oxidase by DHEA requires intact ALCs.

### *Cyp7b1* Is Enriched in Rat ALCs

**Figure 3:**
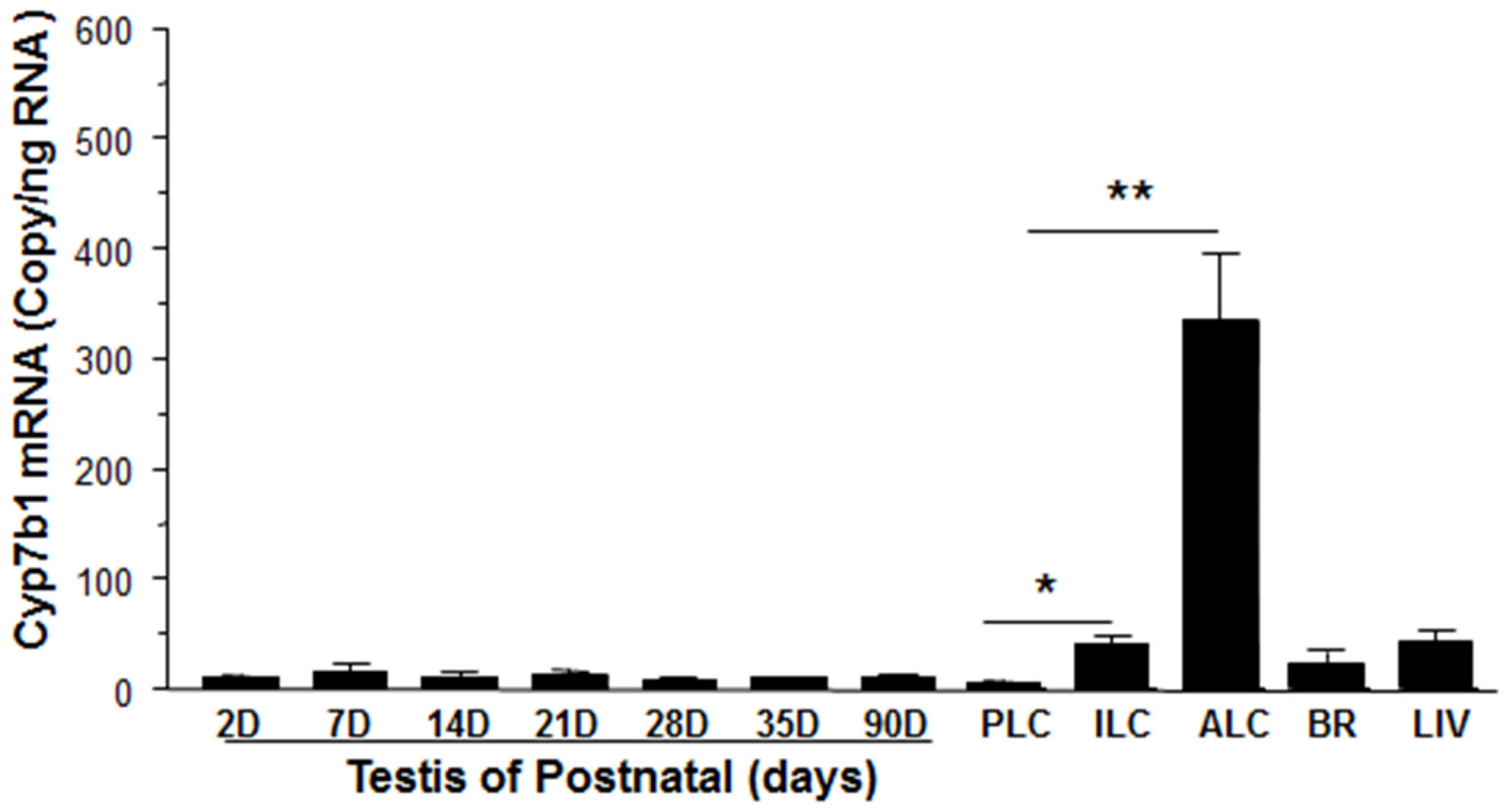
Messenger RNA levels of *Cyp7b1* in postnatal rat testis, brain, and liver as well as purified Leydig cells. Total RNAs from testes at postnatal day (PND) 2 to 90 (2-90D), 90-day-old brain (BR) and liver (LIV), and progenitor (PLCs, isolated from PND21), immature (ILCs, from PND35), and adult Leydig cells (ALCs, from PND90) were isolated, and *Cyp7b1* mRNA levels were measured by qPCR and calculated and adjusted to *Rps16*. Mean ± SEM (*n* = 6). *, ** Indicates significant difference when compared to PLCs at 0.05 and 0.01, respectively.

We determined that the timing of *Cyp7b1* expression in Leydig cells was developmentally relevant. *Cyp7b1* mRNA levels detected by qPCR in templates prepared from rat testis on postnatal days 2–90 did not vary significantly (Figure 3). When Leydig cells were enriched in developmental stages (PLCs at 21 days, ILCs at 35 days, and ALCs at 90 days postpartum), *Cyp7b1* mRNA was 3-folds more abundant in ILCs and 50-folds more abundant in ALCs than that in PLCs (Figure 3). *Cyp7b1* mRNA levels were enriched by 33-folds in ALCs when compared to age-matched testes. This suggests that *Cyp7b1* is primarily expressed in ALCs.

### CYP7B1 Protein Is Present in Rat ALCs

**Figure 4:**
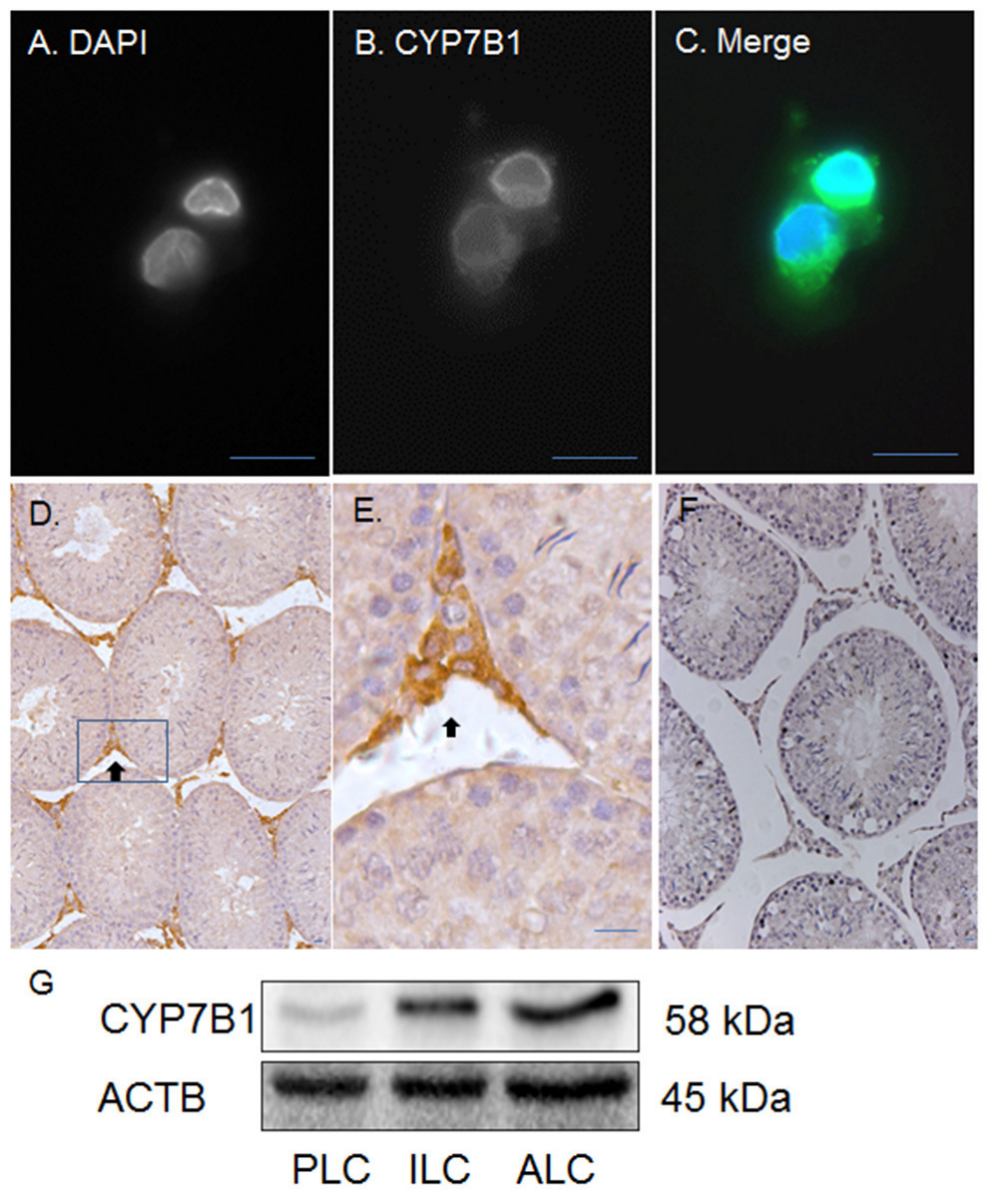
Immunofluorescent and histochemical staining as well as Western blotting of CYP7B1 in rat Leydig cells (LCs) and testis. Polyclonal antibody against CYP7B1 was used. **(A)** is the DAPI contrast staining for nucleus; **(B)** is the fluorescent staining of CYP7B1; **(C)** is the merged picture. CYP7B1 is located in cytoplasmic membrane component. **(D,E)** (magnified inset of **D**) are the CYP7B1 staining in the testis (black arrow). **(F)** is the negative control. **(G)**, Western blotting analysis of CYP7B1, and ACTB in progenitor (PLC), immature (ILC), and adult Leydig cells (ALC). Bar = 10 μm.

Previous studies have demonstrated that CYP7B1 (encoded by *Cyp7b1*) catalyzes DHEA into 7αOHD and that the epimerase activity of 11β-HSD1 converts 7αOHD into 7βOHD (20–22). Besides human, pig and rat 11β-HSD1 can catalyze 7αOHD using NADP+ into 7KD to generate NADPH (23). In order to investigate a potential metabolic coupling between CYP7B1 and 11β-HSD1, we first sought to show whether CYP7B1 was present in rat ALCs. CYP7B1 was detected in ALCs by fluorescent immunohistochemistry and CYP7B1 was present in the cytoplasmic membrane (Figures 4A–E) when compared to the negative control (Figure 4F). Western blotting also showed the highest expression of CYP7B1 in rat ALCs (Figure 4G). This suggests that CYP7B1 is primarily expressed in ALCs.

### 7αOHD Increases 11β-HSD1 Reductase Activity in Intact ALCs

**Figure 5:**
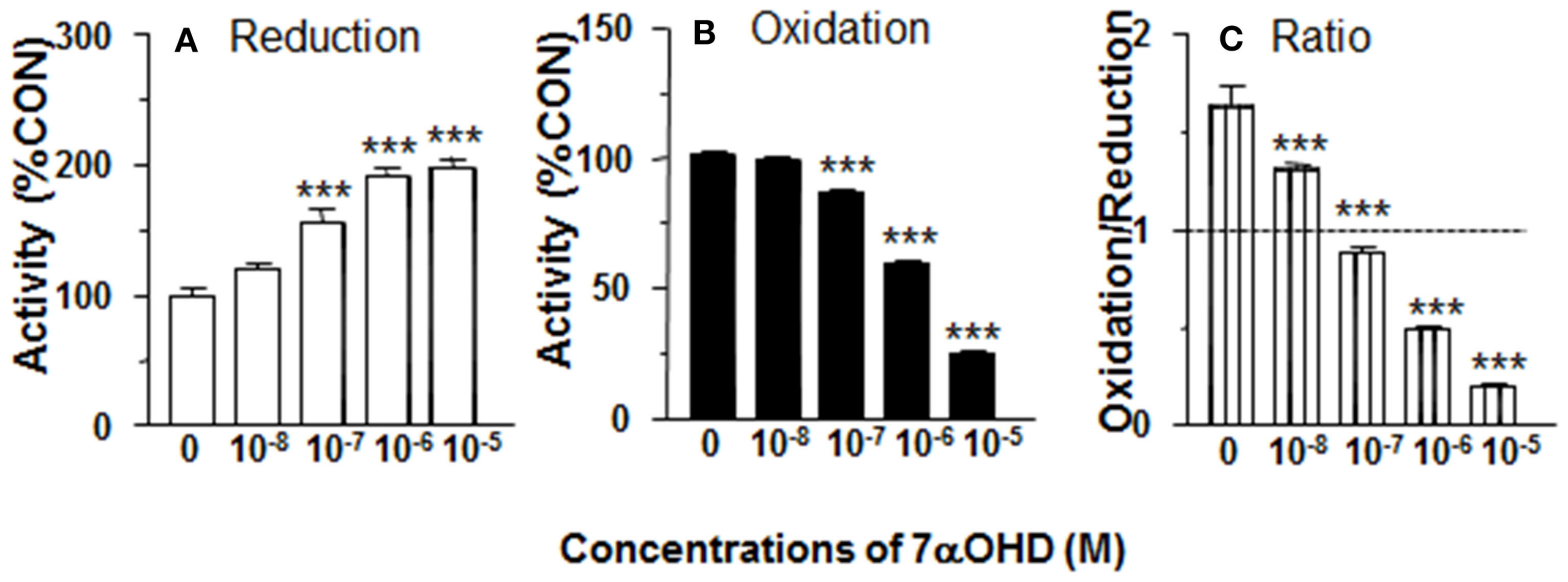
Effects of 7α-hydroxydehydroepiandrosterone (7αOHD) on 11β-HSD1 oxidase and reductase activities in intact adult Leydig cells (ALCs). 2.5 × 10^4^ ALCs were cultured with 25 nM DHC (reductase, **A**) or CORT (oxidase, **B**) for 0.5 h. **(C)** is 11β-HSD1 oxidase/reductase ratio. Mean ± SEM (*n* = 4). * and *** designate significant differences when compared to control (first bar in each panel) at *P* < 0.05 and 0.001, respectively.

CYP7B1 converts DHEA into 7αOHD. 11β-HSD1 reductase activity was measured in intact ALCs in presence of 7αOHD (10 nM−10 μM). 7αOHD increased 11β-HSD1 reductase activity with LOEL of 100 nM (Figure 5A). 7αOHD increased 11β-HSD1 reductase activity to 200% of control at 1 and 10 μM (Figure 5A). The EC_50_ value of 7αOHD of increasing 11β-HSD1 reductase activity was 0.7 μM (Table 1). This suggests that 7αOHD does the opposite action of DHEA on 11β-HSD1 reductase activity in intact ALCs.

### 7αOHD Decreases 11β-HSD1 Oxidase Activity in Intact ALCs

11β-HSD1 oxidase activity was measured in intact ALCs in presence of 7αOHD (10 nM−10 μM). 7αOHD decreased 11β-HSD1 oxidase activity with LOEL of 100 nM (Figure 5B). 7αOHD decreased 11β-HSD1 oxidase activity to 60% of control at 1 μM and 25% of control at 10 μM (Figure 5B). The IC_50_ value of 7αOHD of inhibiting oxidase activity was 1.17 μM (Table 1). We calculated 11β-HSD1 oxidase/reductase ratio (Figure 5C). 7αOHD decreased 11β-HSD1 oxidase/reductase ratio to 50% of control at 1 μM and to 20% of control at 10 μM. This indicates that 7αOHD makes 11β-HSD1 more reductive.

### 7αOHD Increases 11β-HSD1 Reductase Activity in ALC Microsomes

**Figure 6:**
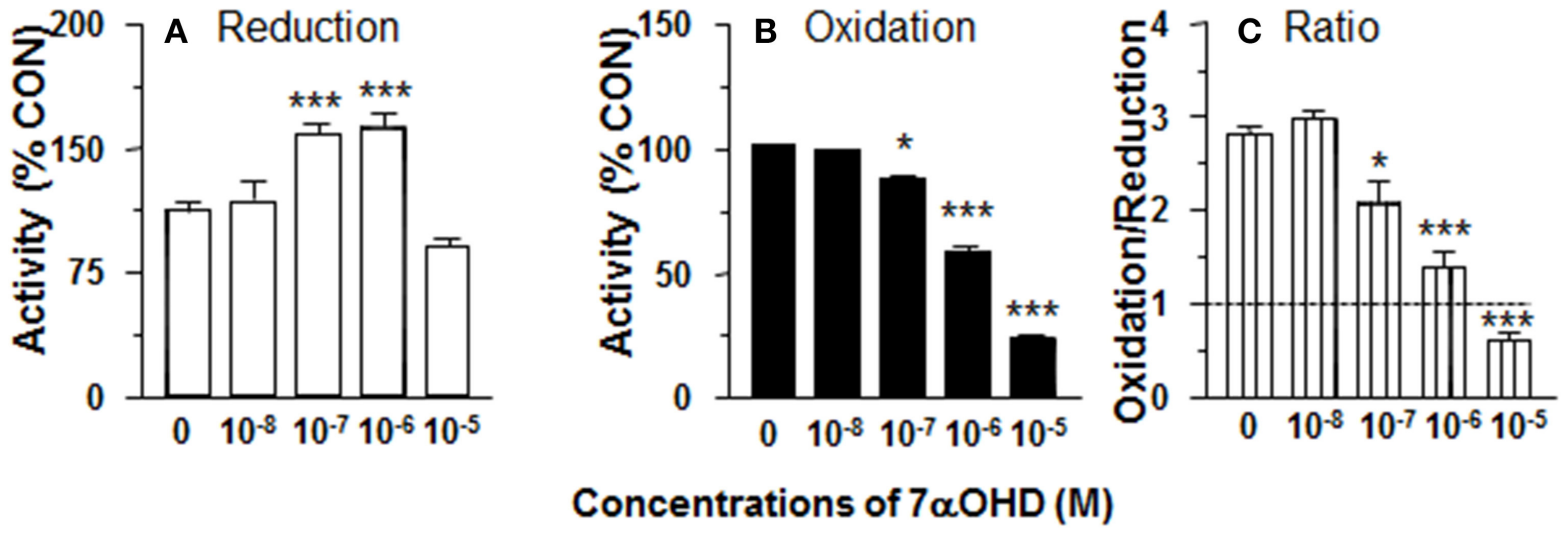
Effects of 7α-hydroxydehydroepiandrosterone (7αOHD) on 11β-HSD1 oxidase and reductase activities in adult Leydig cell (ALC) microsome. 1.5 μg ALC microsome were cultured with 25 nM DHC plus 0.2 mM NADPH (reductase, **A**) or 25 nM CORT plus 0.2 mM NADP+ (oxidase, **B**) for 0.5 h. **(C)** is 11β-HSD1 oxidase/reductase ratio. Mean ± SEM (*n* = 4). * and *** designate significant differences when compared to control (first bar in each panel) at *P* < 0.05 and 0.001, respectively.

11β-HSD1 reductase activity was measured in ALC microsomes in presence of 7αOHD (10 nM-10 μM). 7αOHD increased 11β-HSD1 reductase activity with LOEL of 100 nM. However, at the highest concentration (100 nM), 7αOHD did not affect 11β-HSD1 reductase activity (Figure 6A). The EC_50_ value of 7αOHD of increasing 11β-HSD1 reductase activity was 200 nM (Table 1).

### 7αOHD Inhibits 11β-HSD1 Oxidase Activity in ALC Microsomes

11β-HSD1 oxidase activity was measured in ALC microsomes in presence of 7αOHD (10 nM−10 μM). 7αOHD decreased 11β-HSD1 oxidase activity with LOEL of 100 nM (Figure 6B). 7αOHD decreased 11β-HSD1 oxidase activity to 60% of control at 1 μM and to 25% of control at 10 μM (Figure 6B). The IC_50_ value of 7αOHD for inhibiting oxidase activity was 0.93 μM (Table 1). 7αOHD significantly decreased 11β-HSD1 oxidase/reductase ratio in ALC microsomes at 0.1–10 μM (Figure 6C). The action of 7αOHD in ALC microsomes was similar to that in intact ALCs, indicating that 7αOHD acts on 11β-HSD1 within SER.

### DHEA Is a Competitive Inhibitor of 11β-HSD1 Reductase in ALC Microsomes

**Figure 7:**
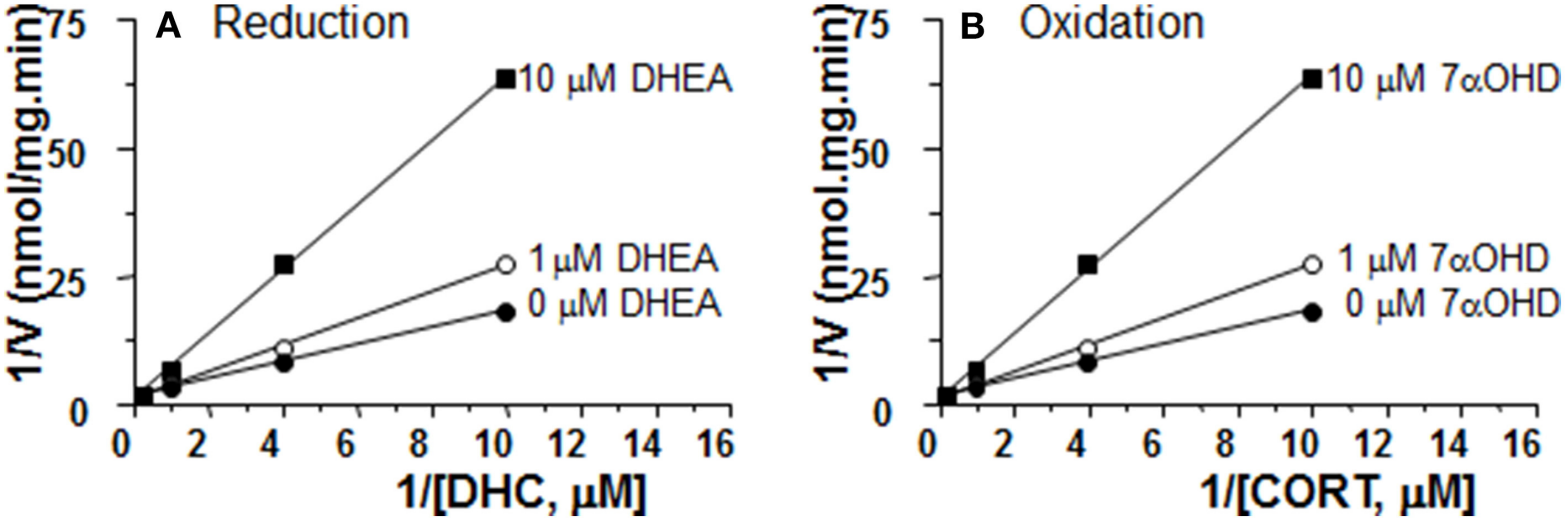
Mode of action for DHEA and 7αOHD of inhibiting 11β-HSD1 activities in adult Leydig cell (ALC) microsomes. Different concentrations of DHC were incubated with 1.5 μg rat ALC microsomes and NADPH for 0.5 h with or without DHEA for 11β-HSD1 reductase **(A)**. Different concentrations of CORT were incubated with 1.5 μg rat ALC microsomes and NADPH for 0.5 h with or without 7αOHD for 11β-HSD1 oxidase **(B)**. Lineweaver-Burk plot for 11β-HSD1 shows a competitive inhibition.

We measured the rates of reductase reactions in ALC microsomes in presence of different DHEA concentrations (0, 1, and 10 μM) in order to evaluate the mode of action. DEHA was a competitive inhibitor of 11β-HSD1 reductase (Figure 7A).

### 7αOHD Is a Competitive Inhibitor of 11β-HSD1 Oxidase in ALC Microsomes

We measured the rates of oxidase reactions in ALC microsomes in presence of different 7αOHD concentrations (0, 1, and 10 μM) in order to evaluate the mode of action. 7αOHD was a competitive inhibitor of 11β-HSD1 oxidase (Figure 7B).

## Discussion

The present study demonstrated that DHEA stimulated 11β-HSD1 oxidase activity and inhibited 11β-HSD1 reductase activity in intact ALCs. The metabolite of DHEA by CYP7B1, 7αOHD, inhibited 11β-HSD1 oxidase activity and stimulated 11β-HSD1 reductase activity in intact ALCs and in ALC microsomes.

CYP7B1 and 11β-HSD1 coexist in rat ALCs. The detectable *Cyp7b1* mRNA and histochemical staining and Western blotting analysis of CYP7B1 indicate that ALCs are the major, if not exclusive, sites of *Cyp7b1* expression. We showed that *Cyp7b1* mRNA levels in purified ALCs were enriched by 33-folds when compared to age-matched testis samples (Figure 3). Developmental increases in *Cyp7b1* mRNA levels were not distinct in the entire testis, but were significant in the purified Leydig cells (Figure 3). Compared to PLCs, *Cyp7b1* mRNA levels were at least 3-folds higher in ILCs and at least 50-folds higher in ALCs (Figure 3). We infer that CYP7B1 is associated with the development and maturation of Leydig cells. Whether LH affects *Cyp17b1* expression is unclear and requires future study. Since *Cyp7b1* is developmentally increased in Leydig cells (Figure 3) and LH is the major factor to regulate Leydig cell development postnatally (30), we speculate that LH can affect *Cyp17b1* expression in rat Leydig cells.

We investigated whether CYP7B1 was coupled with 11β-HSD1. CYP7B1 catalyzes the conversion of DHEA into 7αOHD using NADPH as cofactor (18). The product of NADP^+^ from this reaction might create an environment that accelerates 11β-HSD1 oxidase activity. We tested the hypothesis by adding DHEA to intact ALCs, and found that 11β-HSD1 oxidase activity was significantly increased (Figure 1B). Therefore, it was likely that addition of DHEA drove the formation of NADP^+^. However, the experiment was performed in intact cells. We could not dissect the possible sources of NADP^+^. Therefore, we repeated the experiment by adding DHEA to ALC microsomes, and found that 11β-HSD1 oxidase activity was nearly unchanged (Figure 2B). These results indicate that the stimulation of 11β-HSD1 oxidase activity by DHEA requires cytosolic NADP^+^ pool. Indeed, DHEA has been reported to inhibit glucose-6-phosphate dehydrogenase and increase the cytosolic pool of NADP^+^, which would also drive 11β-HSD1 oxidase activity (31). The present study also showed DHEA was a potent competitive inhibitor of 11β-HSD1 reductase, but not of 11β-HSD1 oxidase. We showed a concentration dependent increase in the ratio of 11β-HSD1 oxidase to reductase (Figure 1C).

7αOHD, the product of DHEA by CYP7B1, is also a substrate for human 11β-HSD1. The conversion of 7αOHD to 7KD by 11β-HSD1 is bidirectional (23). Although human 11β-HSD1 was shown to have epimerase activity, which converts 7αOHD into 7βOHD (32), human, pig and rat 11β-HSD1 can catalyze 7αOHD into KD to generate NADPH (23). 7αOHD was proposed as a native competitor for the 11β-HSD1-mediated activation of cortisone into cortisol (20). However, in the present study, 7αOHD inhibited 11β-HSD1 oxidase and increased 11β-HSD1 reductase, which would have the result of amplifying glucocorticoid actions by converting more cortisone into cortisol (or 11DHC to CORT in rats). 7αOHD was a competitive inhibitor of 11β-HSD1 oxidase (Figure 7B). This is consistent with the finding that 7αOHD shares the active site for cortisone in human 11β-HSD1 (32). The exact mechanism of this inhibition will require further investigation.

The actual physiological impact of 7αOHD is not likely to result in amplification of GC actions. The circulating levels of DHEA sulfate (DHEAS) or DHEAS plus DHEA are much higher than those of cortisol (33–35), these in turn are about three orders of magnitude larger (in humans) than levels of 7αOHD (36, 37). Therefore, among the circulating steroids, DHEA might have more influence than 7αOHD on 11β-HSD1 activities in ALCs, and the oxidase direction will be favored. Although 7αOHD can be generated locally from DHEA by CYP7B1, the relative abundance of CYP7B1 versus 11β-HSD1 will be a factor. In the present study, we found that *Cyp7b1* expression was much lower (300 copy/pg RNA in rat ALCs) compared to 11β-HSD1 mRNA levels in ALCs (500,000 copy/pg RNA) (15). If the mRNA levels are indicative of the actual enzyme levels, it is unlikely that enough 7αOHD will be produced to change the direction of 11β-HSD1

**Figure 8:**
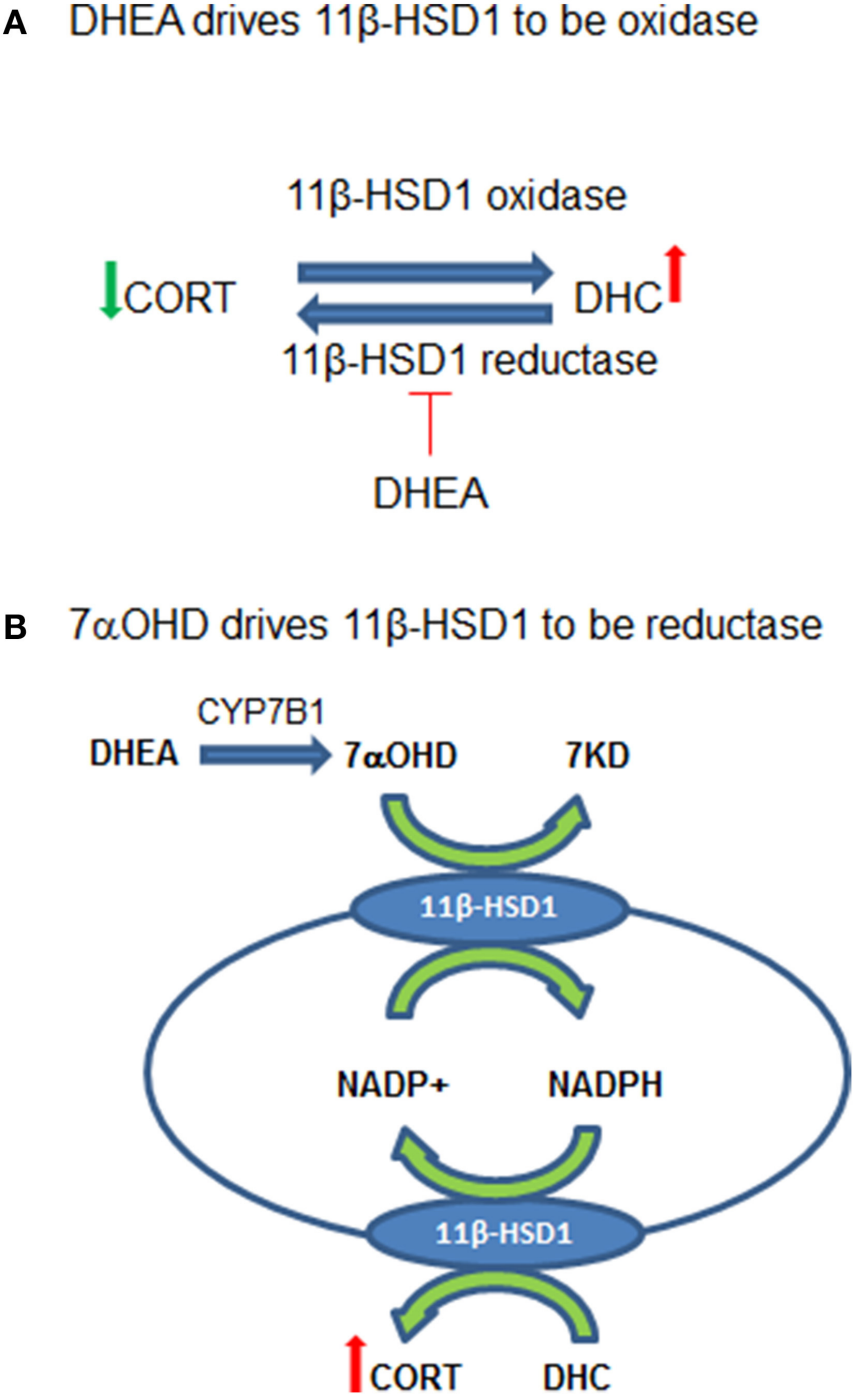
Working hypothesis for the actions of dehydroepiandrosterone (DEHA) and 7α-hydroxydehydroepiandrosterone (7αOHD) in intact rat Leydig cells (ALCs). **(A)** Shows that 11β-hydroxysteroid dehydrogenase 1 (11β-HSD1) is an oxidoreductase in intact ALCs, being oxidase to catalyze corticosterone (CORT) to 11-dehydrocorticosterone (DHC) and being reductase to catalyze DHC to CORT. DHEA inhibits 11β-HSD1 reductase at very low concentrations but does not affect oxidase, thus driving 11β-HSD1 more oxidase. **(B)** Shows that 11β-HSD1 has dual function, catalyzing both 7αOHD and CORT in smooth endoplasmic reticulum. 7αOHD drives 11β-HSD1 toward a reductase. DHEA is converted to 7αOHD in Leydig cells to negatively feedback to counterbalance its action.

In summary, DHEA directly inhibits 11β-HSD1 reductase in the intact ALCs, thus default 11β-HSD1 is enhanced to drive 11β-HSD1 being more oxidative (Figure 8A). CYP7B1 is present in Leydig cells and can catalyze the formation of 7αOHD in ALCs. 7αOHD is the substrate of 11β-HSD1 oxidase, thus competitively inhibiting 11β-HSD1 oxidase to drive 11β-HSD1 being more reductive (Figure 8B). The study is relevant and contribution to endocrinological regulation of testicular GC contents by adrenal steroids. However, the expression of *Cyp7b1* is about 1/1700 of *Hsd11b1* and therefore the action of CYP7B1 may be limited.

## Data Availability Statement

All datasets generated for this study are included in the article/supplementary material.

## Ethics Statement

The animal study was reviewed and approved by the Institutional Animal Care and Use Committee of the Wenzhou Medical University. Written informed consent was obtained from the owners for the participation of their animals in this study.

## Author Contributions

JS and R-SG conceptualized the study design and analyze the data. QZ, YD, XL, CN, and TH performed the experiments and collected the data. R-SG wrote the manuscript and has full access to all the data in the study and takes responsibility for the integrity of the data and the accuracy of the data analysis, and all authors reviewed the manuscript.

### Conflict of Interest

The authors declare that the research was conducted in the absence of any commercial or financial relationships that could be construed as a potential conflict of interest.

## References

[1] Agarwal AK Monder C Eckstein B White PC Cloning and expression of rat cDNA encoding corticosteroid 11beta-dehydrogenase. J Biol Chem. (1989) 264:18939–43. 2808402

[2] Tannin GM Agarwal AK Monder C New MI White PC. The human gene for 11 beta-hydroxysteroid dehydrogenase. Structure, tissue distribution, and chromosomal localization. J Biol Chem. (1991) 266:16653–8. 1885595

[3] Agarwal AK Mune T Monder C White PC. NAD(+)-dependent isoform of 11 beta-hydroxysteroid dehydrogenase. Cloning and characterization of cDNA from sheep kidney. J Biol Chem. (1994) 269:25959–62. 7929304

[4] Albiston AL Obeyesekere VR Smith RE Krozowski ZS. Cloning and tissue distribution of the human 11 beta-hydroxysteroid dehydrogenase type 2 enzyme. Mol Cell Endocrinol. (1994) 105:R11–7. 10.1016/0303-7207(94)90176-7 7859916

[5] Monder C Sakai RR Miroff Y Blanchard DC Blanchard RJ. Reciprocal changes in plasma corticosterone and testosterone in stressed male rats maintained in a visible burrow system: evidence for a mediating role of testicular 11 beta-hydroxysteroid dehydrogenase. Endocrinology. (1994) 134:1193–8. 10.1210/endo.134.3.8119159 8119159

[6] Ge RS Gao HB Nacharaju VL Gunsalus GL Hardy MP. Identification of a kinetically distinct activity of 11beta-hydroxysteroid dehydrogenase in rat Leydig cells. Endocrinology. (1997) 138:2435–42. 10.1210/endo.138.6.5165 9165033

[7] White PC Mune T Rogerson FM Kayes KM Agarwal AK 11β-Hydroxysteroid dehydrogenase and its role in the syndrome of apparent mineralocorticoid excess. Pediatr Res. (1997) 41:25–9. 10.1203/00006450-199701000-00004 8979285

[8] Draper N Walker EA Bujalska IJ Tomlinson JW Chalder SM Arlt W. Mutations in the genes encoding 11β-hydroxysteroid dehydrogenase type 1 and hexose-6-phosphate dehydrogenase interact to cause cortisone reductase deficiency. Nat Genet. (2003) 34:434–9. 10.1038/ng1214 12858176

[9] Atanasov AG Nashev LG Schweizer RA Frick C Odermatt A. Hexose-6-phosphate dehydrogenase determines the reaction direction of 11β-hydroxysteroid dehydrogenase type 1 as an oxoreductase. FEBS Lett. (2004) 571:129–33. 10.1016/j.febslet.2004.06.065 15280030

[10] Bujalska IJ Draper N Michailidou Z Tomlinson JW White PC Chapman KE. Hexose-6-phosphate dehydrogenase confers oxo-reductase activity upon 11 beta-hydroxysteroid dehydrogenase type 1. J Mol Endocrinol. (2005) 34:675–84. 10.1677/jme.1.01718 15956339

[11] Banhegyi G Csala M Benedetti A. Hexose-6-phosphate dehydrogenase: linking endocrinology and metabolism in the endoplasmic reticulum. J Mol Endocrinol. (2009) 42:283–9. 10.1677/JME-08-0156 19060178

[12] Hewitt KN Walker EA Stewart PM. Minireview: hexose-6-phosphate dehydrogenase and redox control of 11{beta}-hydroxysteroid dehydrogenase type 1 activity. Endocrinology. (2005) 146:2539–43. 10.1210/en.2005-0117 15774558

[13] Lavery GG Walker EA Turan N Rogoff D Ryder JW Shelton JM. Deletion of hexose-6-phosphate dehydrogenase activates the unfolded protein response pathway and induces skeletal myopathy. J Biol Chem. (2008) 283:8453–61. 10.1074/jbc.M710067200 18222920 PMC2417187

[14] Li X Hu G Wang YY Hu YY Zhou H Latif SA. Metabolic coupling determines the activity: comparison of 11β-hydroxysteroid dehydrogenase 1 and its coupling between liver parenchymal cells and testicular leydig cells. PLoS ONE. (2015) 10:e0141767. 10.1371/journal.pone.0141767 26528718 PMC4631333

[15] Ge RS Dong Q Niu EM Sottas CM Hardy DO Catterall JF. 11{beta} -Hydroxysteroid dehydrogenase 2 in rat leydig cells: its role in blunting glucocorticoid action at physiological levels of substrate. Endocrinology. (2005) 146:2657–64. 10.1210/en.2005-0046 15761036

[16] Imamichi Y Yuhki KI Orisaka M Kitano T Mukai K Ushikubi F. 11-Ketotestosterone is a major androgen produced in human gonads. J Clin Endocrinol Metab. (2016) 101:3582–91. 10.1210/jc.2016-2311 27428878

[17] Gao HB Ge RS Lakshmi V Marandici A Hardy MP. Hormonal regulation of oxidative and reductive activities of 11 beta-hydroxysteroid dehydrogenase in rat Leydig cells. Endocrinology. (1997) 138:156–61. 10.1210/endo.138.1.4837 8977399

[18] Martin C Bean R Rose K Habib F Seckl J. cyp7b1 catalyses the 7alpha-hydroxylation of dehydroepiandrosterone and 25-hydroxycholesterol in rat prostate. Biochem J. (2001) 355:509–15. 10.1042/bj3550509 11284740 PMC1221764

[19] Rose K Allan A Gauldie S Stapleton G Dobbie L Dott K. Neurosteroid hydroxylase CYP7B: vivid reporter activity in dentate gyrus of gene-targeted mice and abolition of a widespread pathway of steroid and oxysterol hydroxylation. J Biol Chem. (2001) 276:23937–44. 10.1074/jbc.M011564200 11290741

[20] Muller C Pompon D Urban P Morfin R. Inter-conversion of 7alpha- and 7beta-hydroxy-dehydroepiandrosterone by the human 11beta-hydroxysteroid dehydrogenase type 1. J Steroid Biochem Mol Biol. (2006) 99:215–22. 10.1016/j.jsbmb.2005.12.001 16603347

[21] Hennebert O Pernelle C Ferroud C Morfin R. 7alpha- and 7beta-hydroxy-epiandrosterone as substrates and inhibitors for the human 11beta-hydroxysteroid dehydrogenase type 1. J Steroid Biochem Mol Biol. (2007) 105:159–65. 10.1016/j.jsbmb.2006.11.021 17624766

[22] Niro S Hennebert O Morfin R. A native steroid hormone derivative triggers the resolution of inflammation. Horm Mol Biol Clin Investig. (2010) 1:11–9. 10.1515/hmbci.2010.001 25961967

[23] Robinzon B Michael KK Ripp SL Winters SJ Prough RA. Glucocorticoids inhibit interconversion of 7-hydroxy and 7-oxo metabolites of dehydroepiandrosterone: a role for 11beta-hydroxysteroid dehydrogenases? Arch Biochem Biophys. (2003) 412:251–8. 10.1016/S0003-9861(03)00056-0 12667489

[24] Lakshmi V Monder C. Extraction of 11 beta-hydroxysteroid dehydrogenase from rat liver microsomes by detergents. J Steroid Biochem. (1985) 22:331–40. 10.1016/0022-4731(85)90435-2 3857394

[25] Shan LX Hardy MP. Developmental changes in levels of luteinizing hormone receptor and androgen receptor in rat Leydig cells. Endocrinology. (1992) 131:1107–14. 10.1210/endo.131.3.1505454 1505454

[26] Salva A Klinefelter GR Hardy MP. Purification of rat leydig cells: increased yields after unit-gravity sedimentation of collagenase-dispersed interstitial cells. J Androl. (2001) 22:665–71. 11451364

[27] Payne AH Wong KL Vega MM. Differential effects of single and repeated administrations of gonadotropins on luteinizing hormone receptors and testosterone synthesis in two populations of Leydig cells. J Biol Chem. (1980) 255:7118–22. 6248547

[28] Mo J Chen X Ni C Wu K Li X Zhu Q. Fibroblast growth factor homologous factor 1 stimulates Leydig cell regeneration from stem cells in male rats. J Cell Mol Med. (2019) 23:5618–31. 10.1111/jcmm.14461 31222931 PMC6653537

[29] Wu X Guo X Wang H Zhou S Li L Chen X. A brief exposure to cadmium impairs Leydig cell regeneration in the adult rat testis. Sci Rep. (2017) 7:6337. 10.1038/s41598-017-06870-0 28740105 PMC5524795

[30] Ye L Li X Li L Chen H Ge RS. Insights into the development of the adult Leydig cell lineage from stem leydig cells. Front Physiol. (2017) 8:430. 10.3389/fphys.2017.00430 28701961 PMC5487449

[31] Mccormick KL Wang X Mick GJ. Evidence that the 11 beta-hydroxysteroid dehydrogenase (11 beta-HSD1) is regulated by pentose pathway flux. Studies in rat adipocytes and microsomes. J Biol Chem. (2006) 281:341–7. 10.1074/jbc.M506026200 16234247

[32] Hennebert O Montes M Favre-Reguillon A Chermette H Ferroud C Morfin R. Epimerase activity of the human 11beta-hydroxysteroid dehydrogenase type 1 on 7-hydroxylated C19-steroids. J Steroid Biochem Mol Biol. (2009) 114:57–63. 10.1016/j.jsbmb.2008.12.015 19167490

[33] Touitou Y Sulon J Bogdan A Touitou C Reinberg A Beck H. Adrenal circadian system in young and elderly human subjects: a comparative study. J Endocrinol. (1982) 93:201–10. 10.1677/joe.0.0930201 7086322

[34] Waltman C Blackman MR Chrousos GP Riemann C Harman SM. Spontaneous and glucocorticoid-inhibited adrenocorticotropic hormone and cortisol secretion are similar in healthy young and old men. J Clin Endocrinol Metab. (1991) 73:495–502. 10.1210/jcem-73-3-495 1651956

[35] Sulcova J Hill M Hampl R Starka L. Age and sex related differences in serum levels of unconjugated dehydroepiandrosterone and its sulphate in normal subjects. J Endocrinol. (1997) 154:57–62. 10.1677/joe.0.1540057 9246938

[36] Lapcik O Hampl R Hill M Starka L. Immunoassay of 7-hydroxysteroids: 2. Radioimmunoassay of 7alpha-hydroxy-dehydroepiandrosterone. J Steroid Biochem Mol Biol. (1999) 71:231–7. 10.1016/S0960-0760(99)00145-4 10704912

[37] Cooper MS Syddall HE Fall CH Wood PJ Stewart PM Cooper C. Circulating cortisone levels are associated with biochemical markers of bone formation and lumbar spine BMD: the Hertfordshire cohort study. Clin Endocrinol. (2005) 62:692–7. 10.1111/j.1365-2265.2005.02281.x 15943831

